# Rheological Measurements and Structural Analysis of Polymeric Materials

**DOI:** 10.3390/polym13071123

**Published:** 2021-04-01

**Authors:** Helmut Münstedt

**Affiliations:** Department of Materials Science and Engineering, Friedrich-Alexander-University Erlangen-Nuremberg, D-91058 Erlangen, Germany; helmut.muenstedt@fau.de

**Keywords:** rheological properties, polymer melts, relations with molecular parameters, analysis of branching content, mechanical pretreatment effect, studies of thermal stability, particle-filled polymeric systems, rheological behavior of heterogeneous polymers

## Abstract

Rheological measurements of polymer melts are widely used for quality control and the optimization of processing. Another interesting field of rheology is to provide information about molecular parameters of polymers and the structure build-up in heterogeneous polymeric systems. This paper gives an overview of the influence of molar mass, molar mass distribution and long-chain branching on various rheological characteristics and describes the analytical power following from established relations. With respect to applications, we discuss how rheological measurements can be used to gain insight into the thermal stability of a material. A special impact lies in the demonstration, how long-chain branching can be analyzed using rheological means like the zero-shear viscosity as a function of molar mass and strain hardening occurring in elongation. For contributions to branching analysis, the thermorheological behavior and activation energies are particularly discussed. The use of elastic quantities in the case of mechanical pretreatment effects is briefly addressed. The influence of fillers on recoverable properties in the linear range of deformation is analyzed and the role of their specific surface area for interactions described. It is shown how the fundamental results can be applied to study the state of nanoparticle dispersions obtained under special conditions. Furthermore, it is demonstrated that the findings on polymer/filler systems are the base of structure analyses in heterogeneous polymeric materials like polyvinylchloride (PVC) and acrylonitrile–butadiene–styrene copolymers (ABS).

## 1. Introduction

Rheological investigations on polymer melts serve two main purposes. On one hand, they are used to support the processing of polymers; on the other, from rheological properties, insights can be obtained into some features of molecular structure. These relations can contribute to tailoring polymer materials for distinct applications, but additionally, they can be employed to qualitatively follow up the change of molecular parameters under external parameters like heat and mechanical deformation that may occur during various processing operations. Furthermore, some rheological properties may support the analysis of polymers because of their high sensitivity concerning molecular quantities.

For dispersed polymeric systems like particle-filled materials and polymer blends, rheological measurements could be applied as an easy method to qualitatively investigate interactions between different phases and changes of geometric structures built up by the heterogeneities.

The role of rheology for structural analysis has been relatively little addressed, and that is the reason for the Special Issue, “Rheology as a Tool for the Investigation of Structures of Polymeric Materials” in the journal “Polymers”. This paper is intended to throw some light on several aspects of this topic, which have been discussed in detail in corresponding articles by various authors.

## 2. Rheological Methods

The mechanical behavior of polymer melts is rather complex because they are viscoelastic materials, and, consequently, their properties are time-dependent. Furthermore, most polymers exhibit a distinct nonlinear behavior, which is particularly pronounced at the conditions of processing. Moreover, viscoelastic properties can be strongly affected by temperature and hydrostatic pressure. Another feature of polymer melts is that their elongation behavior cannot be generally derived from that in shear, but both deformation modes play a role in processing.

Due to the power of macromolecular chemistry, the chemical composition of polymers is very variable, and alterations of the length, the topographical structure and the distributions of various molecules contribute to the great variety of products. The variability is still enlarged by the possibility to blend species with each other or to add fillers of different kinds.

This paper mainly deals with linear properties in shear because they are very suitable for analytical purposes. Many methods to determine rheological characteristics have been established over the years, and their scopes were described and discussed in various textbooks on rheology. Therefore, only the essentials supporting an easy reading of this paper are shortly mentioned. Often [1] is cited here for fundamentals, but other references would provide comparable information.

### 2.1. Investigations in the Linear Regime

Rheological measurements in the linear range of deformation have the advantages that they can be more easily and more completely described by theories than those in the nonlinear regime. Such studies are very efficient to support the structural analysis of polymers and, thus, play the dominant role in this paper.

#### 2.1.1. Dynamic-Mechanical Measurements

**Figure 1:**
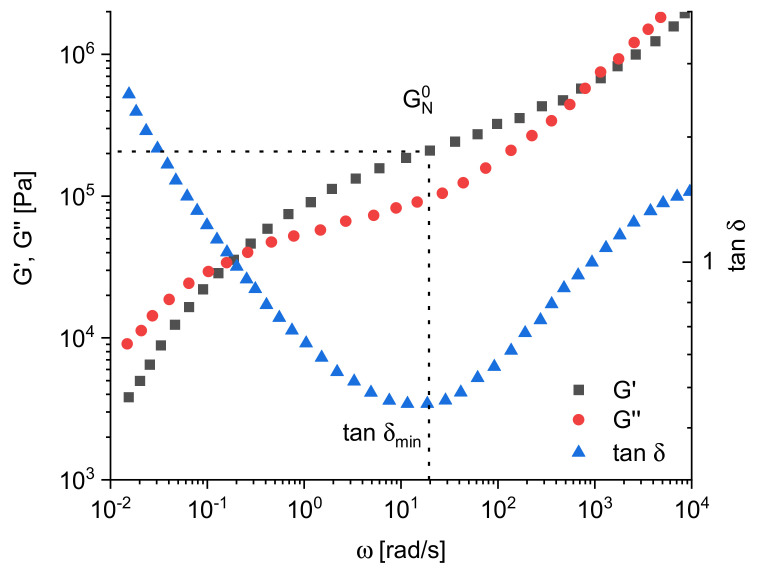
Example for storage modulus *G*′, loss modulus *G*″, and tangent of the phase angle *δ* as functions of the angular frequency *ω* (polyisobutylene with $M_{w}$ = 85 kg/mol and $M_{w}/M_{n}$ = 2) [2].

A very versatile method for the characterization of polymer melts is dynamic mechanical measurement. Several instruments are commercially available, and their highly developed hardware and software are the reason for being widely used today in laboratory routine. Figure 1 gives an example for the most usual presentation of dynamic mechanical measurements. At low angular frequencies in the so-called terminal regime, the imaginary part *G*″ and the real part *G*′ of the complex modulus G* = *G*′ + *iG*″ follow the well-known relations:

(1) $$\log\;G\prime \prime =\;\log\;\eta_{0}+\;\log\;\omega$$

and

(2) $$\log\;G\prime \;=\;\log\;\eta_{0}^{2}\;J_{e}^{0}\;+\;2\;\log\;\omega$$

with *η*_0_ being the zero-shear viscosity and $J_{e}^{0}$ the linear steady-state recoverable compliance (e.g., [1]). As shown further down in this paper, these quantities play an essential role in molecular analysis. *η*_0_ can be obviously determined according to Equation (1) and $\eta_{0}^{2}J_{e}^{0}$ according to Equation (2). $J_{e}^{0}$ follows then from the quotient *G*′/*G*”^2^ in the terminal regime.

As shown exemplarily in Figure 1, *G*′ and *G*″ increase with *ω* and exhibit a distinct crossover point. Considering the Maxwell model for a description of the frequency dependence of the moduli, the angular frequency *ω*_c_ at the crossover point of *G*′(ω) and *G*″(ω) corresponds to the longest relaxation time:

(3) $$\tau_{e}\;=\;\eta_{0}J_{e}^{0}=\;1/\omega_{c}$$

which is discussed by some authors as a characteristic quantity.

For some analytical applications, the phase angle *δ* related to the moduli by tan *δ* = *G*″/*G*′ is used. As seen from Figure 1 for polymer melts, tan *δ* decreases with increasing frequency first and then runs through a distinct minimum.

Another interesting rheological quantity following from dynamic mechanical measurements is the plateau modulus $G_{N}^{0}$ defined as the frequency-independent storage modulus. Applying the theory of rubber elasticity, an entanglement molar mass $M_{e}$ corresponding to the molar mass of strands between the crosslinks of rubbery material can be derived according to:

(4) $$G_{N}^{0}\;=\;\frac{4}{5}\frac{\rho RT}{M_{e}}$$

*ρ* is the density, *T* the absolute temperature and *R* the universal gas constant. Alternatively, other prefactors are used in the literature. However, for thermoplastic materials, a distinct plateau is only found for narrowly distributed polymers with polydispersity indices $M_{w}/M_{n}$ around 1. For most polymers and particularly those used for technical applications, *G*′(ω) shows a behavior like the polyisobutylene in Figure 1. In such cases, $G_{N}^{0}$ is approximated by *G*′ at the frequency of the minimum of tan *δ* as sketched in Figure 1. In [2], different methods for the determination of $G_{N}^{0}$ are discussed and their accuracies compared. The frequency dependency of *G*′ can become of special analytical interest for particle-filled polymer melts for which the formation of a network may occur.

For a great number of polymeric materials, viscosity functions, which play an important role in processing, can be derived from dynamic mechanical measurements making use of the Cox–Merz rule. This relation says that the magnitude of the complex viscosity |η*| = |G*|/*ω* as a function of the angular frequency ω is equivalent to *η*($\dot{\gamma }$), where $\dot{\gamma }$ is the shear rate (e.g., [1]).

#### 2.1.2. Creep and Creep Recovery Experiments

A very versatile method to determine rheological properties in the linear regime is creep experiments and the subsequent recovery, which reveals the elastic portion of the previous deformation. Creep recovery is particularly useful when long experimental times are necessary to attain a steady state of deformation. Again, the principles of such measurements have been described in textbooks on rheology. A discussion of their special features can be found in [3].

The appropriate rheological quantities for presenting creep recovery experiments are the creep compliance:

*J* = *γ*/*σ* (5)

with *γ* being the shear deformation under the constant shear stress *σ* and the recoverable shear compliance:

*J_r_* = *γ_r_*/*σ* (6)

where *γ_r_* describes the recoverable portion of the previous total deformation at zero stress. From the linear theory of viscoelasticity follows for the time dependence of the creep compliance:

*J*(*t*) = *J*_0_ + *Ψ*(*t*) + *t*/*η*_0_ = *J_r_*(*t*) + *t*/*η*_0_ (7)

with *J*_0_ being the momentary elastic compliance, *Ψ*(*t*) the so-called creep function and $\eta_{0}$ the zero-shear viscosity. According to Boltzmann’s superposition principle, the creep function can be numerically described by a discrete spectrum of retardation times *τ_i_* and retardation strengths *J_i_* according to:

(8) $$\Psi \left(t_{r}\right)=\sum_{i=1}^{n}J_{i}\;(1-\;e^{-t_{r}/\tau_{i}})$$

and for $t_{r}\;\gg \;\tau_{i}$, the linear steady-state recoverable compliance follows as:

(9) $$J_{e}^{0}=\underset{t_{r}\to \infty }{\lim}J_{r}\left(t_{r}\right)=J_{0}\;\;+\sum_{i=1}^{n}J_{i}$$

For polymer melts, *J*_0_ is comparably small and can be often neglected. For the matter of completeness, it should be mentioned that the discrete spectrum may be replaced by a continuous spectrum, and then instead of the sum in Equation (8), an integral over the retardation times is used.

From the creep compliance, the zero-shear viscosity *η*_0_ can be determined according to Equation (7) if the creep time *t* is chosen so long that the finite steady-state value of the creep function can be neglected in comparison to *t*/*η*_0_. This state is indicated by the slope of 1 in a double-logarithmic plot of *J*(*t*). From the intercept of the straight line or more conveniently from a plot of *t*/*J*(*t*) versus *t* the viscosity can be determined [3].

### 2.2. Investigations in the Nonlinear Regime

In comparison to the power of rheological measurements in the linear range, those in the nonlinear regime play a minor role in structural analysis. Nevertheless, some of them are frequently used to obtain a quick qualitative insight into molecular changes.

#### 2.2.1. Capillary Rheometry

Extruding a polymer melt through a capillary of definite geometry is a straightforward method to study the flow behavior of polymer melts. Thus, capillary rheometers are widely used to measure viscosity functions in a range of shear rates occurring in processing operations. The melt index determined by a standardized simple extrusion procedure has developed over the years into a well-accepted method for quality control. The swell of a strand at the exit of a capillary is sometimes used for a rough assessment of elastic properties of a polymer melt because it reflects the recoverable portions of the deformation the melt has experienced passing barrel and die of a capillary rheometer.

#### 2.2.2. Extensional Rheometry

A strongly nonlinear deformation mode for many polymer melts is stretching in laboratory rheometers. Experimental methods and special features are comprehensively discussed in [1], for example. The elongational behavior in the nonlinear range cannot be derived from that in shear and, thus, it is understandable why some effects of the molecular structure show up very distinctly in the elongational viscosity, for example. The elongational viscosity is defined as:

(10) $$\mu \;=\;\sigma_{E}/\dot{\varepsilon }$$

with *σ*_E_ being the tensile stress and $\dot{\varepsilon }$ the elongational rate.

Besides viscous properties, the elastic behavior in elongation can be measured by letting the elongated sample recover in any state of extension. The recoverable Hencky strain follows as:

(11) $$\varepsilon_{r}\;=\;\ln\lambda_{r}\;=\;\ln l/l_{r}$$

where $\lambda_{r}$ is the recoverable stretching ratio, l the length of the elongated sample and $l_{r}$ the length after recovery.

Some devices allow the visual observation of the sample during deformation. Of special interest with respect to structural changes during deformation is the possibility of the Münstedt tensile rheometer (MTR) (see [1]) to freeze the sample in any state of extension and study the morphology of polymer blends, for example, with suitable methods outside the rheometer.

## 3. Rheological Properties and Molecular Structure

The rheological properties of polymers are markedly determined by their molecular structure. Because most of the polymeric materials are composed of molecules of different lengths, their distributions are commonly characterized by the weight-average molar mass *M_w_* and the polydispersity index *M_w_*/*M_n,_* with *M_n_* being the number average molar mass. The definitions of these quantities and their analytical determination can be found in various textbooks. For applications, branched molecules play an important role, and particularly long-chain branches have an influence on the processing and end-use properties of polymeric materials. Long-chain branches are defined by molar masses larger than the molar mass between molecule entanglements, and, thus, it is obvious that an influence on rheological properties can be expected. Some polymeric species are modified by short-chain branches, which do not much affect the rheological behavior but are of advantage for certain end-use properties.

### 3.1. Zero-Shear Viscosity

**Figure 2:**
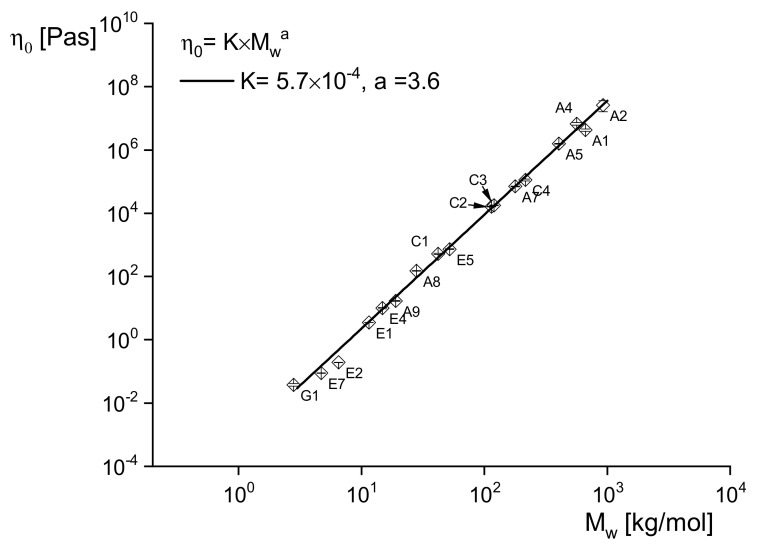
Zero-shear viscosity $\eta_{0}$ at 150 °C for several linear polyethylenes of various weight-average molar masses $M_{w}$ and molar mass distributions [4].

The zero-shear viscosity *η*_0_ plays an important role in the structural analysis of polymeric materials insofar as for linear materials above a critical molar mass $M_{c}$ the well-known relationship

(12) $$\eta_{0}∼\;M_{w}^{a}$$

holds. For the exponent, *a*, values between 3.4 and 3.8 have been reported in the literature. Figure 2 demonstrates on linear polyethylenes of a wide range of weight-average molar masses between 3 and 900 kg/mol how well this relation is fulfilled with *a* = 3.6 independently of the molar mass distribution characterized by *M_w_*/*M_n_* ranging from 2.9 to 16. Similar relations are reported in the literature for other polymers.

The significant power-law dependence of $\eta_{0}$ on *M_w_* has an interesting consequence for analytical purposes: A slight change of *M_w_* has a great effect on $\eta_{0}$ and, thus, makes it a sensitive tool to probe molecular alterations due to processing or handling and to investigate the thermal stability of a polymer. Having a relation like that above for linear polyethylenes in hand, rheological measurements can even determine *M_w_*. For most polymeric materials, it needs much experimental care to determine $\eta_{0}$, however, because the linear range and the steady-state must be attained. However, it is shown later that rheological studies based on Equation (12) have important practical relevance.

**Figure 3:**
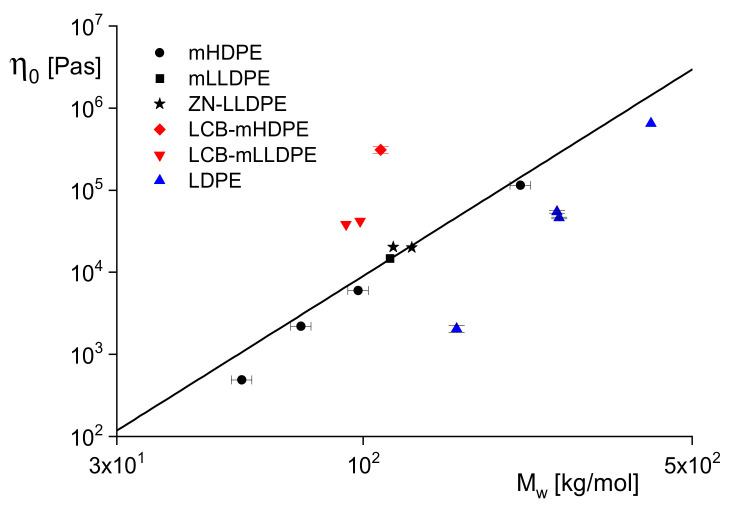
Zero-shear viscosity $\eta_{0}$ at 150 °C as a function of the weight-average molar mass $M_{w}$ for linear and long-chain branched polyethylenes polymerized along different routes [5]. The full line represents the power-law according to Equation (12) with the exponent *a* = 3.4.

Whereas the relation between $\eta_{0}$ and $M_{w}$ is considered being independent of the molar mass distribution, there are significant dependencies on long-chain branching, as becomes obvious from Figure 3. The linear sample mHDPE and the short-chain branched mLLDPE were polymerized with a metallocene catalyst, the short-chain branched ZN-LLDPE with a Ziegler-Natta catalyst. Their data come to lie on one curve and are in line with the widely accepted knowledge that short-chain branched LLDPEs and linear polyethylenes of different molar mass distributions follow the same power-law. The LDPEs with a tree-like branching structure have smaller viscosities than the linear samples; the long-chain branched metallocene polyethylenes LCB–mHDPE and LCB–mLLDPE with a star-like branching structure lie above the power-law line. These results are confirmed in the literature and, thus, can be used for a qualitative assessment of branching architectures.

### 3.2. Linear Steady-State Recoverable Compliance

#### 3.2.1. Dependence on $M_{w}$ and $M_{w}/M_{n}$

**Figure 4:**
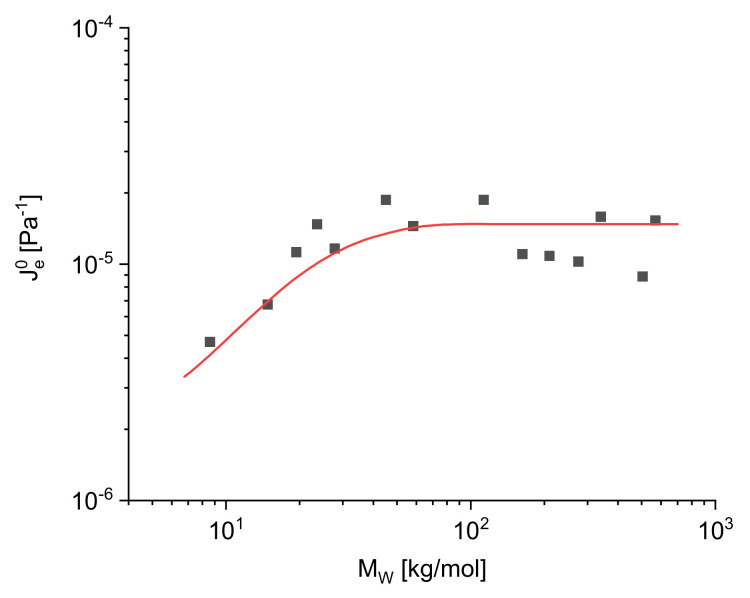
Linear steady-state recoverable compliance $J_{e}^{0}$ as a function of the weight-average molar mass $M_{w}$ for various anionic polystyrenes [6].

**Figure 5:**
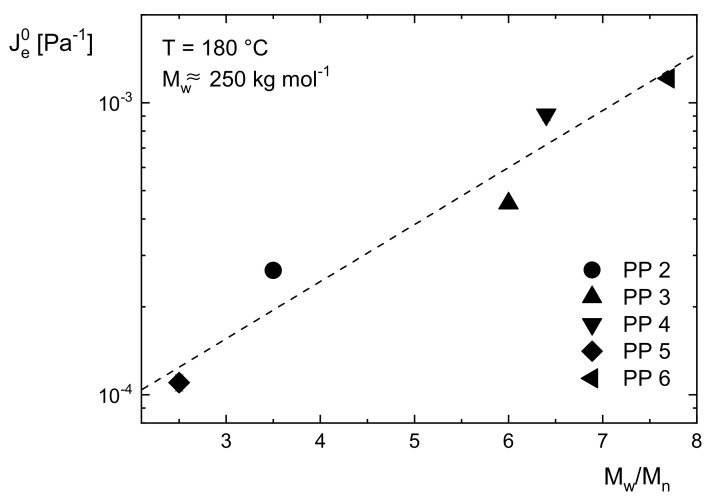
Linear steady-state recoverable compliance $J_{e}^{0}$ as a function of the polydispersity index $M_{w}/M_{n}$ for five commercial polypropylenes with different molar masses [7]. The line is drawn to guide the eye.

While for linear polymers, the zero-shear viscosity as a function of the weight-average molar mass is independent of the molar mass distribution, for the linear steady-state recoverable compliance $J_{e}^{0}$ it is just the other way round, as may be concluded from Figure 4 and Figure 5. In Figure 4, $J_{e}^{0}$ is presented for different anionic polystyrenes with

a narrow molar mass distribution of $M_{w}/M_{n}\approx 1$ and molar masses $M_{w}$ ranging from 10 to 500 kg/mol. Up to about 50 kg/mol, $J_{e}^{0}$ increases with $M_{w},$ but above a critical molar mass $M_{c}$ it becomes independent of the molar mass within the accuracy of the measurements. $M_{c}$ is about three times the entanglement molar mass $M_{e}$. In Figure 5, $J_{e}^{0}$ is shown for five commercial polypropylenes with different molar masses distinctly above the critical value $M_{c}$ and various polydispersity indices between 2 and 8. $J_{e}^{0}$ clearly increases with $M_{w}/M_{n}$. This result shows that $J_{e}^{0}$ can be used as a sensitive indicator for the molar mass distribution. However, a general numerical relation between $J_{e}^{0}$ and quantities used for the description of molar mass distributions has not been established up to now as discussed in [8]. Thus, the line is only drawn to guide the eye.

The determination of $J_{e}^{0}$, either from creep recovery experiments as shown as an example in Figure 18 for PMMA filled with nanoparticles or from dynamic mechanical measurements according to Section 2.2.1, needs much experimental care. Qualitative hints to changes in the elasticity of a polymer melt can be obtained from measurements of the extrudate swell in capillary experiments or the recoverable portion of elongation (e.g., [8]). Both experiments are performed in the nonlinear regime, however. Particularly, the qualitative determination of the extrudate swell can conveniently be carried out with a capillary rheometer or even with a simple melt indexer and may be used as a coarse method for checking the molar mass distribution for quality control, for example.

#### 3.2.2. Influence of Branching

**Figure 6:**
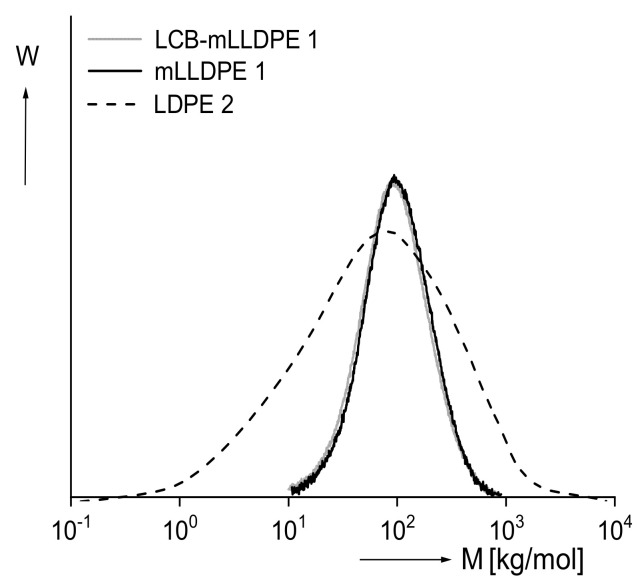
Molar mass distribution of a commercial low-density polyethylene (LDPE) in comparison with a long-chain branched linear low-density polyethylene (LCB–mLLDPE) that was polymerized with a metallocene catalyst. The mLLDPE is a short-chain branched sample with butene as a comonomer [9].

**Figure 7:**
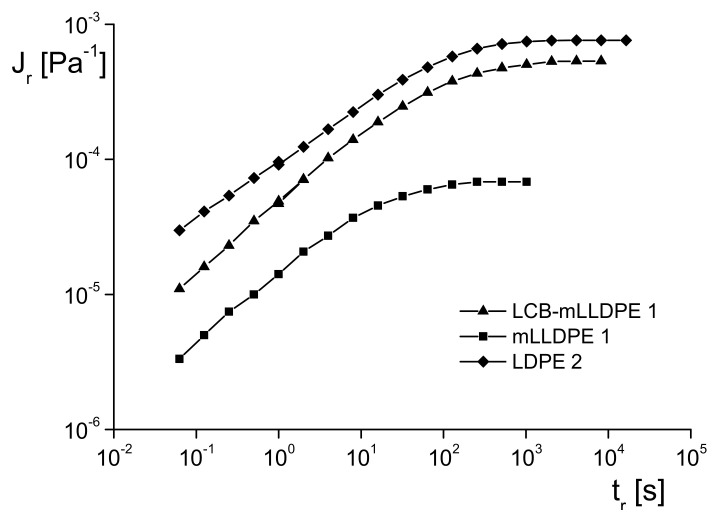
Recoverable compliance $J_{r}$ in the linear range as a function of the recovery time $t_{r}$ for the mLLDPE, LCB–mLLDPE and LDPE characterized in Figure 6 [9].

An answer to the question of how elastic properties are influenced by branching and in which way they could be used for information about the molecular structure is not straightforward because the obvious effect of the molar mass distribution must be separated from that of branches. An indication of the complexity becomes exemplarily obvious from the studies presented in Figure 6 and Figure 7 on various polyethylenes. Figure 6 exhibits the molar mass distributions of the linear mLLDPE and the star-like branched LCB–mLLDPE, both polymerized with metallocene catalysts and that of a classical LDPE with a tree-like branching structure. The LDPE has a much broader molar mass distribution than the two other samples that are not distinguishable from each other within the accuracy of their measurement.

For the three polyethylenes, the recoverable compliances $J_{r}$ in the linear range of deformation are presented as a function of the recovery time $t_{r}$ in Figure 7. All the samples reach a pronounced steady state of recovery. The linear polyethylene mLLDPE exhibits the lowest recoverable compliance over the whole range of recovery times applied. The long-chain branched sample with the same narrow molar mass distribution shows a distinct higher recoverable compliance. From this finding, it can be concluded that long-chain branches increase the elasticity of polyethylene. The LDPE exhibits a still more pronounced recoverable compliance over the whole range of recovery times, but because the molar mass distribution is so much broader than that of the mLLDPE and LCB–mLLDPE, the branching effect cannot even be estimated. Thus, the elastic behavior of polymer melts is not very suitable for support of the branching analysis.

### 3.3. Thermorheological Behavior and Activation Energy

The temperature dependence of many rheological properties of polymer melts is determined by a special relation between time and temperature in the way that the input of thermal energy increases the mobility of macromolecule segments and shortens the duration of relaxation processes. When all the times of a relaxation or retardation spectrum, respectively, are changed by temperature, in the same way, the time dependencies of rheological properties at different temperatures can be normalized by altering the experimental times relevant for the particular quantity by a temperature-dependent factor. On a logarithmic scale, this corresponds to a shift of curves along their time-related axes by the shift factor. In this way, a so-called master curve is obtained. Polymers for which master curves exist are called thermorheologically simple. The fundamentals of this behavior and its importance for polymer physics and rheology are discussed in several textbooks.

**Figure 8:**
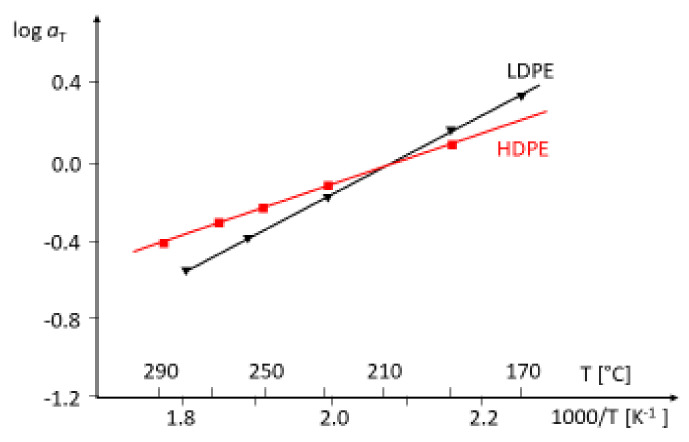
Arrhenius plots of an HDPE and an LDPE. The reference temperature is $T_{0}=210\;{}^{\circ}C$.

A special role for the topic of this paper plays the temperature dependence of the shift factor in the case of melts at temperatures distinctly above the glass transition. For homopolymers of this kind, it is well known that the shift factors $a_{T}$ as functions of the absolute temperature T follow the Arrhenius equation:

(13) $$a_{T}\;=\;\exp\;\left[\frac{E_{a}}{R}\left(\frac{1}{T}-\;\frac{1}{T_{0}}\right)\right]$$

and, thus, the activation energy $E_{a}$, which is typical of a species, can be determined. *R* is the universal gas constant and $T_{0}$ the reference temperature. In Figure 8, the Arrhenius diagrams of an HDPE and an LDPE are shown. For the LDPE, an activation energy higher than that of the HDPE follows. The most common method to obtain master curves is the shift of dynamic mechanical measurements at different temperatures. This procedure is rather time-consuming and, thus, the activation energy plays only a minor role in the rheological polymer analysis.

Interestingly, some conclusions can be drawn for materials, which do not fulfill the time–temperature superposition principle. They are called thermorheologically complex. Thermorheological complexity formally occurs when the relaxation times of a sample do not have the same temperature dependence. The consequences are that an Arrhenius diagram may be only attainable for a chosen value of the loss or storage modulus, for example. Consequently, the activation energy is not a unique material property any longer but becomes a function of the particular rheological quantity. Pioneering work in this field is reported in [10]. The physical reason for such a behavior can be that the sample consists of molecules of species, which have different temperature dependencies as in a polymer blend, for example. Another reason could be the existence of molecules with various molecular architectures, as it may occur in metallocene polymerized polyethylene, where linear and branched molecules can be present.

An elegant way to qualitatively study whether material is thermorheologically simple or complex is a plot of data from dynamic mechanical experiments proposed by van Gurp and Palmen [11]. They considered the directly measured phase angle *δ* as a function of the magnitude of the complex modulus |G*| = $\sigma_{0}/\gamma_{0}$ where $\gamma_{0}$ and $\sigma_{0}$ are the amplitudes of the oscillatory shear and stress, respectively. Phase angle and magnitude of the modulus are functions of the angular frequency. In the case of the validity of the time–temperature superposition principle, the two quantities can be shifted with the same factor $a_{T}$ along the ω axis to get a master curve when the temperature is changed. Thus, the frequency dependency can be eliminated and *δ*(|$G^{*}$|) becomes independent of temperature. Consequently, for shift factors depending on the dynamic-mechanical quantity chosen, *δ*(|$G^{*}$|) is not independent of temperature any longer. Such behavior indicates thermorheological complexity.

**Figure 9:**
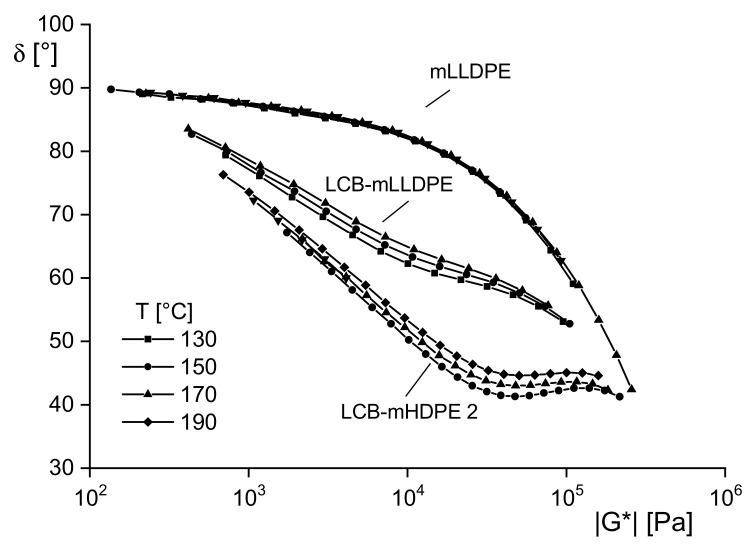
Phase angle *δ* as a function of the magnitude of the complex modulus |*G* *| for the linear polyethylene mLLDPE and the two long-chain branched polyethylenes LCB-mLLDPE and LCB-mHDPE at various temperatures [5].

Figure 9 shows *δ*(|$G^{*}$|) of three polyethylenes at different temperatures. For the linear mLLDPE, the curves at the four temperatures fall together. The conclusion of thermorheological simplicity of this type of material agrees with the experience from the frequency shift of dynamic mechanical data. The plots for the long-chain branched polyethylenes polymerized by metallocene catalysts differ for the various temperatures and indicate thermorheological complexity. These results are in agreement with molecular analyses that show the existence of linear and branched molecules that have different activation energies as well-known. Considering these findings in the light of structural analysis, dynamic-mechanical measurements presented as van Gurp–Palmen plots are an elegant method to get information on the thermorheological behavior of a polymeric material that can be used for qualitative insights into its branching structure.

Obviously, shift factors and activation energies cannot be obtained from a van Gurp–Palmen plot. For this purpose, the time or frequency functions of particular rheological quantities at various temperatures must be evaluated. In [10], it was shown that activation energies could be obtained by slice-wise shifts of dynamic-mechanical properties as functions of angular frequency measured at different temperatures. This method promises some structural insights based on activation energies even for thermorheologically complex materials, and it was used to analyze various long-chain branched polyethylenes in [12]. The phase angle *δ* was shown to be particularly suitable for shifting because obviously, the density correction with temperature has not to be applied, which is necessary for a precise shift of the moduli.

The potential of a quantitative evaluation of the thermorheologically complex behavior with respect to relations between modulus-dependent activation energies $E_{a}$ and molecular structures has not been widely used up to now due to the somewhat elaborate procedure for the determination of $E_{a}$. In [13], a numerical method is presented for the calculations of activation energies in terms of dependence on the moduli *G*′, *G*″ or the phase angle *δ*. For this purpose, the moduli as a function of angular frequency ω measured at several temperatures are described by polynomials. Then the angular frequencies at preselected values of the moduli or *δ* are calculated from the curves at different temperatures, and for these data, Arrhenius plots are set up, from which activation energies are determined. These activation energies can be finally discussed in terms of dependence on the dynamic-mechanical quantity chosen. As a special feature of long-chain branched polyethylenes, it was found that $E_{a}\left(\delta \right)$ runs through a maximum, whereas the activation energies, in terms of dependence on the moduli, are descending functions.

The maximum values $E_{a}^{max}$ and their positions $\delta_{max}$ are discussed in [14] for blends of long-chain branched metallocene-catalyzed polyethylenes with different molar mass distributions. The measurements clearly demonstrate the experimental difficulties of this method and the uncertainties to determine the characteristic data. More fundamental work must be done to establish activation energies in terms of dependence on dynamic mechanical properties for thermorheologically complex polymers as a reliable rheological tool for the support of branching analysis.

## 4. Examples of Practical Applications

Based on the fundamental relations between rheological properties and molecular structure discussed above, some simple methods useful for applications of polymers were derived. For example, one point of interest is material stability during processing operations and how long it might be exposed to certain thermal conditions before being degraded too much. Other useful applications have evolved from measurements of rheological properties that sensitively reflect structural elements like long-chain branches.

### 4.1. Thermal Stability

A very important molecular parameter is the weight-average molar mass $M_{w}$. As mentioned above, it is very sensitively related to the zero-shear viscosity $\eta_{0}$ by the power 3.4. On the other side, $\eta_{0}$ determines the frequency dependence of the loss modulus G″ at low frequencies and $\eta_{0}^{2}$ that of the storage modulus *G*′ (see Equations (1) and (2)).

Thus, in the terminal regime

(14) $$G\prime /\omega^{2}=\;J_{e}^{0}\eta_{0}^{2}\;\approx M_{w}^{6.8}$$

is valid for commercial products because $J_{e}^{0}$ is independent of the molar mass (see Figure 4). This relation is obviously extremely sensitive with respect to changes in the molar mass. *G*′ can conveniently be measured by commercial rotational rheometers even at low frequencies. From these facts follows a very useful method to trace a molar mass change: For a frequency, which should be selected reasonably low, *G*′ is registered as a function of time. Even in the case that the chosen frequency lies somewhat outside the validity of Equation (14), high sensitivity concerning the molar mass is still available.

**Figure 10:**
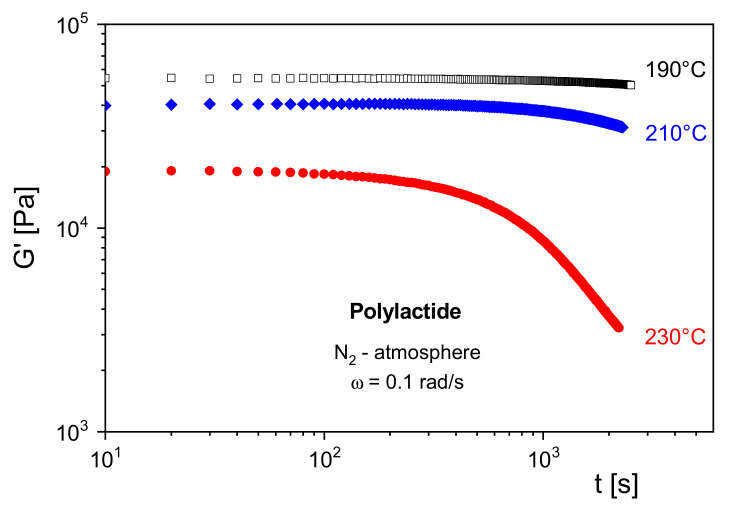
Storage modulus *G*′ as function of the residence time *t* in the rheometer for a polylactide.at several temperatures.

In Figure 10, *G*′ of a polylactide (PLA) is shown at several temperatures as a function of the residence time in a rotational rheometer. Under nitrogen atmosphere, *G*′ remains constant at 190 °C for the chosen time of 3000 s. At 210 °C, a decrease of the modulus becomes visible around 1000 s, and at 230 °C, *G*′ starts to decline already around 100 s. From the measurements, a thermally induced decrease of the molar mass can be concluded, which is due to depolymerization. Furthermore, it may be derived that for processing operations above 210 °C special care must be taken considering the residence time of the PLA in the machines.

**Figure 11:**
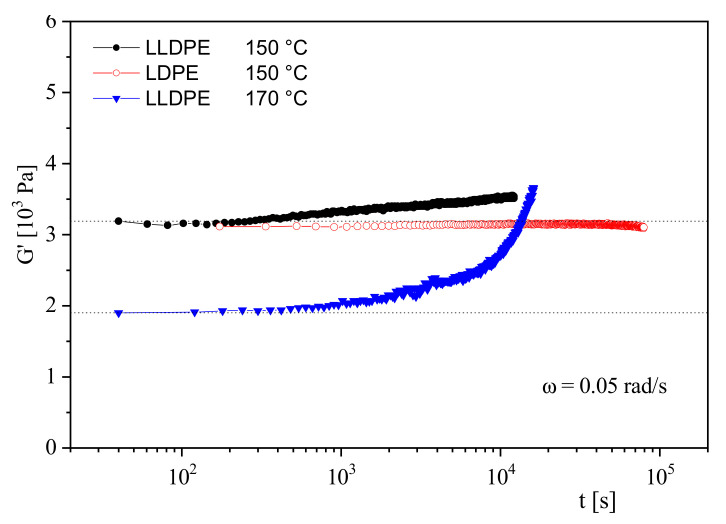
Storage modulus *G*′ as function of residence time *t* in the rheometer for a low-density polyethylene (LDPE) and a linear low-density polyethylene (LLDPE) at two temperatures.

Figure 11 gives an example of the thermal stability of an LDPE and an LLDPE. At 150 °C, *G*′ of the LDPE does not change over a long time of 80,000 s, whereas the modulus of the LLDPE at this temperature starts to rise already at 200 s. At 170 °C, the modulus increase is much steeper. The growing modulus indicates a surge in molar mass that could be induced by a partial crosslinking of molecules. In practice, the thermal stability of polymers is often improved by the addition of stabilizers. Their efficiency can be tested and optimized by the simple rheological measurement presented in the Figure 10 and Figure 11.

**Figure 12:**
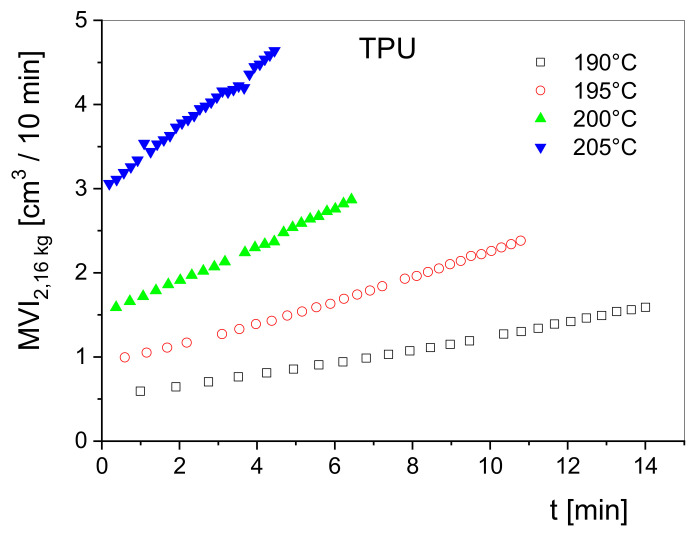
Melt index *MVI* as a function of the residence time *t* in the barrel of a melt indexer for a thermoplastic polyurethane (TPU) at several temperatures. The piston load was 2.16 kg and the preheating time about 3 min.

Simple rheological stability tests can be performed using easy-to-handle devices like a melt indexer. Here, the volume rate of a melt flowing through a capillary at a fixed piston load is measured as a function of the residence time in the barrel of the apparatus. From throughput curves at various temperatures, the thermal stability can be qualitatively assessed. This method is less sensitive than measuring *G*′ at low frequencies because the throughput is related to viscosities at shear rates that are often somewhat larger than those for attaining the Newtonian regime and, thus, the dependence on molar mass is weaker than that of $\eta_{0}$. Measurements of this kind can be very useful, however, as shown in Figure 12. Here, the volume rate at a piston load of 2.16 kg conventionally measured in cm^3^/10 min obtained from a melt indexer is plotted as a function of the residence time in the barrel of the device for a thermoplastic polyurethane at several temperatures. The measurements show the high-temperature sensitivity of the material and provide a rough overview of its degradation rates. Obviously, the degradation rate distinctly increases with temperature and indicates a thermally activated decay of the macromolecules. Studies of this kind can support insights into molecular reactions and contribute to the optimization of processing such heat-sensitive materials.

Because measurements of the melt index are widely applied in quality control, it should be mentioned that in cases where a suitable strand at the die exit can be obtained, its swell could be used for getting a qualitative insight into the development of the molar mass distribution of a polymer after various treatments or during exposure to heat. It is well established that an increase of extrudate swell can be related to a broader molar mass distribution (see [1], for example).

### 4.2. Analysis of Long-Chain Branches

Another example of the support of structural analysis using rheological experiments is related to long-chain branches (LCB), which play an important role in several engineering plastics. The usual way to characterize long-chain branched polymers is to measure the intrinsic viscosity or the radius of gyration of the molecules as a function of the molar masses within a sample. These techniques use viscosity measurements or multi-angle laser light scattering (MALLS), respectively, coupled with gel permeation chromatography. Detailed information can be found in several corresponding textbooks.

**Figure 13:**
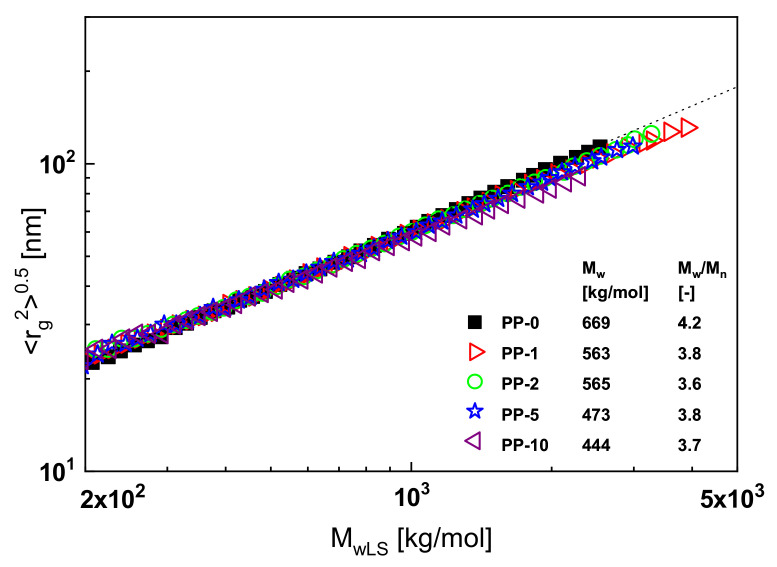
Root of the mean-square radius of gyration <*r*_g_^2^>^0.5^ as a function of the absolute molar mass *M_wLS_* determined by light scattering for the linear polypropylene PP-0 and the samples PP-1 to PP-10 irradiated with doses from 1 to 10 kGy.

An example of such a classical branching analysis is shown in Figure 13. Here, the radius of gyration presented as the root of its mean square is plotted as a function of the absolute value of the molar mass for polypropylene samples exposed to electron irradiation of various intensities. The intensities measured in kGy correspond to the numbers of the sample designations. In a double-logarithmic plot, the measured radii of gyration as a function of the absolute molar masses *M_wLS_* determined by laser light scattering of the non-irradiated linear polypropylene PP-0 follow the broken straight line that represents the Mark–Houwink equation for PP according to the literature. Long-chain branches decrease the radii of gyration in comparison to linear molecules of the same molar mass, and this effect is more pronounced the more effective the branches are (for details, see [1], for example). In Figure 13, all the irradiated samples, with the exception of PP-10, lie on the line for the linear PP within the accuracy of the measurements. From this result, it may be concluded that an irradiation dose of 10 kGy is necessary to create a measurable amount of long-chain branches. The inset contains further information on molecular parameters. It shows that the molar mass decreases with irradiation and the polydispersity index becomes slightly smaller.

**Figure 14:**
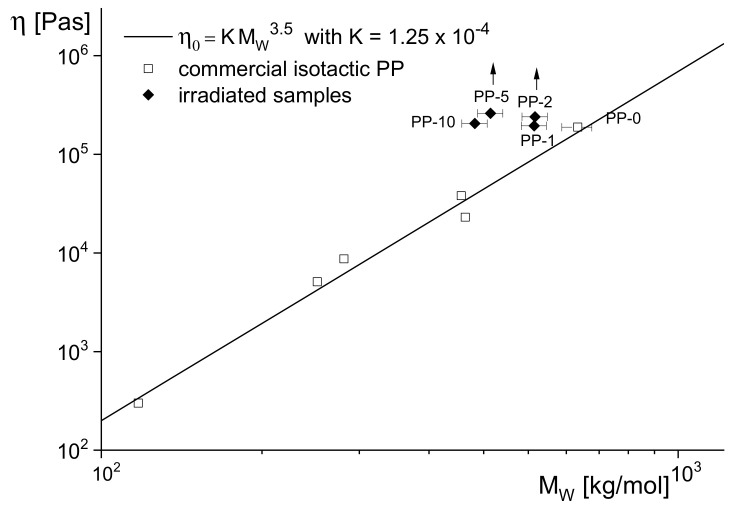
Zero-shear viscosities $\eta_{0}$ at 180 °C in dependence on absolute molar masses M_w_ for several linear PP and the irradiated samples PP-1, PP-2, PP-5 and PP-10 [15]. The arrows indicate that the steady state was not reached.

However, rheological measurements demonstrate that at the lower doses, long-chain branches are already existent. In Figure 14, the zero-shear viscosities in terms of dependence on the absolute molar masses are shown for the irradiated samples together with the well-established power-law for commercial linear PPs. All irradiated samples lie significantly above the reference line of the linear products, and the arrows for PP-2 and PP-5 indicate still higher viscosities due to the fact that for these samples, the Newtonian region was not reached under the experimental conditions applied.

From the zero-shear viscosities lying above the curve for the linear polypropylenes, additional information on the molecular structure can be derived. As shown in [16] for various types of polyethylenes and demonstrated in Figure 3, samples with star-like long-chain branches have viscosities higher than those of their linear counterparts of the same molar mass, but those of a tree-like architecture exhibit lower values. The comprehensive analysis of polypropylenes irradiated with various doses up to 150 kGy has been reported in [15], and it looks reasonable from this study that the weakly irradiated PP samples have a star-like branching structure.

**Figure 15:**
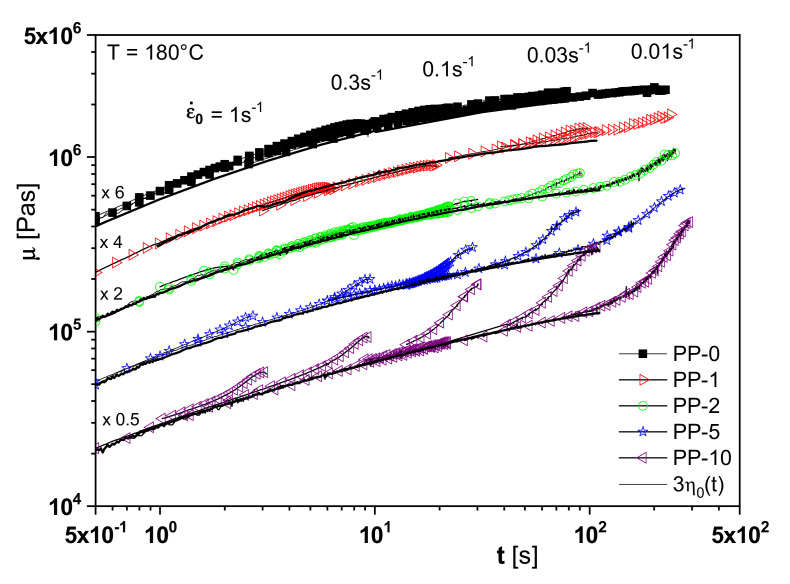
Elongational viscosities *µ* at 180 °C and various elongation rates $\dot{\varepsilon }$ of the polypropylenes of Figure 13 and Figure 14, electron-beam irradiated with various doses [15]. The full lines represent the three-fold time-dependent zero-shear viscosities $\eta_{0}\left(t\right)$ and confirm the validity of Trouton’s relation. For a better distinction, the curves are shifted along the viscosity axis with respect to PP-5 by the factors indicated.

The branching analysis of the irradiated polypropylenes is supported by another rheological property, namely extensional viscosity. One feature of the elongational behavior is the increase of the extensional viscosity with time, the so-called strain hardening, for long-chain branched polymers or species with a broad molar mass distribution or a definite high molar mass component (see [1], for example,). As follows from Figure 15, PP-2 already shows a distinct strain hardening compared to the non-irradiated linear PP-0. This effect is more pronounced for PP-5 and PP-10 and points to an increasing branching efficiency. This study on irradiated polypropylenes is an example of how sophisticated rheological experiments can be applied to improve branching analysis.

### 4.3. Rheological Measurements as a Tool to Study the Mechanical Pretreatment Effect

**Figure 16:**
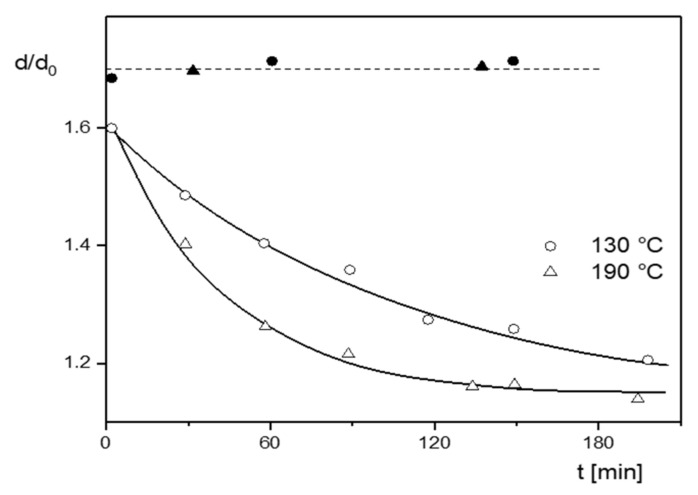
Swelling ratio *d*/*d*_0_ of an LDPE with *M_w_* = 500 kg/mol as a function of the kneading time *t* in a Brabender plastograph at two kneading temperatures (open symbols) and after dissolution in xylene and subsequent precipitation (closed symbols). *d* is the diameter of the extrudate and *d_0_* that of the die of the melt indexer used [19].

For many years it has been known that a mechanical pretreatment of polymer melts can be used to improve their processing behavior and some end-use properties. For example, for long-chain branched polyethylenes, it has been reported in [17] that the extensibility and optical transparency of blown films improved when the polymeric material had been previously sheared in a special laboratory extruder. These results were confirmed by the work of other authors [18], and it was found that simple rheological properties like melt index and extrudate swell could be used to characterize the pretreatment effect (e.g., [19]). The melt index increased, and the extrudate swell decreased upon mechanical pretreatment, and these changes were found to be reversible after heat treatment or the dissolution in a solvent and subsequent precipitation of the polymer. This result is interesting insofar as it shows that a molar mass degradation during the mechanical treatment can be excluded as the reason for the effect observed, but that it must be sought in reversible alterations of interactions of the macromolecules. In Figure 16, measurements of the extrudate swell of an LDPE by Rokudai are shown, from which some essential features of the pretreatment effect become obvious [19]. The extrudate swell decreases first with kneading time in a Brabender plastograph and then approaches a plateau. The effect of kneading on the sample is more pronounced at the higher temperature, which means the underlying structural changes are thermally activated. The dissolved and precipitated samples exhibit a significant increase of extrudate swell up to a value that is independent of the previous kneading time and is in agreement with the untreated material. This reversibility is a distinct hint to molecular parameters remaining unchanged by the shear applied.

The structural mechanisms underlying the mechanical pretreatment effect are still a matter of discussion, but nevertheless, rheological measurements have shown to be an efficient tool to discover essential features of this interesting property.

## 5. Rheological Properties and Dispersed Polymeric Systems

Besides contributions to the molecular structure analysis of homopolymers, rheological measurements can provide insights into heterogeneous polymeric systems. Of importance for practical applications are polymer blends with immiscible purely organic phases or particle-filled polymers, where the second phase consists of an inorganic material. The kind, size, and geometry of organic and inorganic heterogeneities cover a wide range and, moreover, the different contents used influence material properties. At higher concentrations, the secondary phases can form agglomerates that may affect the material performance. Nanosized particles are of special interest with respect to rheological properties because of their large specific surface area and the resulting strong interactions with matrix molecules. The rheological behavior of a polymer melt with nanoparticles is exemplarily discussed in the following.

### 5.1. Influence of Nanoparticle Concentration

**Figure 17:**
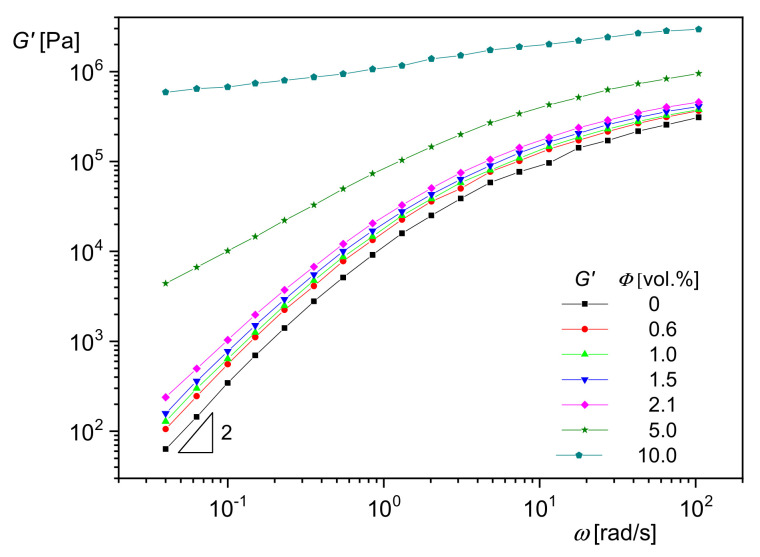
Storage modulus *G*′ as a function of angular frequency *ω* for various concentrations of silica particles with the average diameter *d*_50_ = 20 nm in polymethylmethacrylate (PMMA) at 200 °C [20].

In Figure 17, the effect of silica nanoparticles with an average diameter of *d*_50_ = 20 nm on the storage modulus *G*′ as a function of the angular frequency *ω* is shown for polymethylmethacrylate (PMMA) as the matrix. *G*′(*ω*) increases with particle concentration, and the slope, which is typically 2 for melts of homopolymers in the terminal regime, becomes smaller. Both effects indicate a growing influence of the particles on the mobility of the PMMA molecules. At the highest concentration of 10 vol%, the modulus approaches a plateau independent of frequency. Such a behavior is typical of polymers with a network penetrating the sample as found for crosslinked species like rubber. In the case of particle-filled systems, it can be assumed that the fillers are able to form agglomerates that hinder the mobility of matrix molecules.

**Figure 18:**
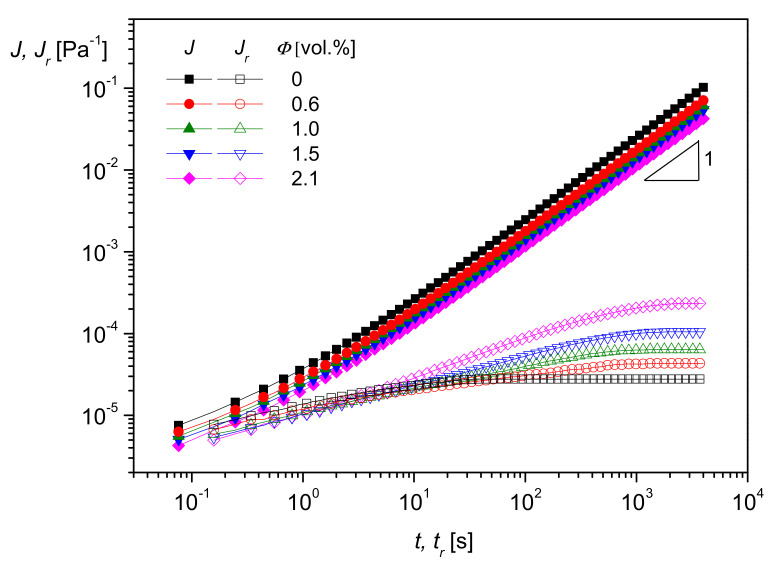
Creep compliance J and creep recovery compliance $J_{r}$ as functions of creep time *t* and recovery time $t_{r}$, respectively, at the stress *σ* = 50 Pa and 200 °C for PMMA and several concentrations of nanosilica with the average particle diameter *d*_50_ = 20 nm [20].

A very interesting method to rheologically characterize filled polymer melts and to get an idea of interactions between particles and matrix molecules is creep recovery experiments. An example of PMMA filled with the silica nanoparticles of Figure 17 is shown in Figure 18. The creep compliance *J* and the recoverable creep compliance *J_r_* are plotted as functions of creep time *t* and recovery time *t_r_*, respectively, at the creep stress *σ* = 50 Pa in the linear regime of deformation.

As can be seen, log *J*(*t*) increases linearly with log *t* at longer creep times, and, thus, the zero-shear viscosity $\eta_{0}$ may be determined according to Section 2.1.2 from the intercept. As follows from Figure 18, and it is well-known for particle-filled polymer melts, $\eta_{0\;}$ increases with filler content and using creep experiments, quantitative relations may be established [21]. The recoverable compliance $J_{r}$ attains a plateau as a function of the recovery time *t_r_*, and its value corresponds to the linear steady-state compliance $J_{e}^{0}$. According to Figure 18, this quantity characteristic of the elastic properties of a polymer melt increases with the volume concentration of nanosilica measured up to 2.1 vol%. As described in [21], from $J_{r\;}\left(t_{r}\right)$ retardation spectra can be calculated using Equation (8). They show that a pronounced retardation time occurs in the spectrum of the material when nanoparticles are added and that the retardation strengths increase with particle content. Analyses of this kind are the base of models that support an understanding of properties and help to develop materials for special applications. In [21], a model is presented where matrix molecules are attached to the filler surface, and their mobility is reduced by these physical interactions. This model was verified by its ability to describe various properties of the PMMA/nanosilica system at least qualitatively.

### 5.2. Influence of Nanoparticle Size on Creep Recovery and Studies of Dispersion States

**Figure 19:**
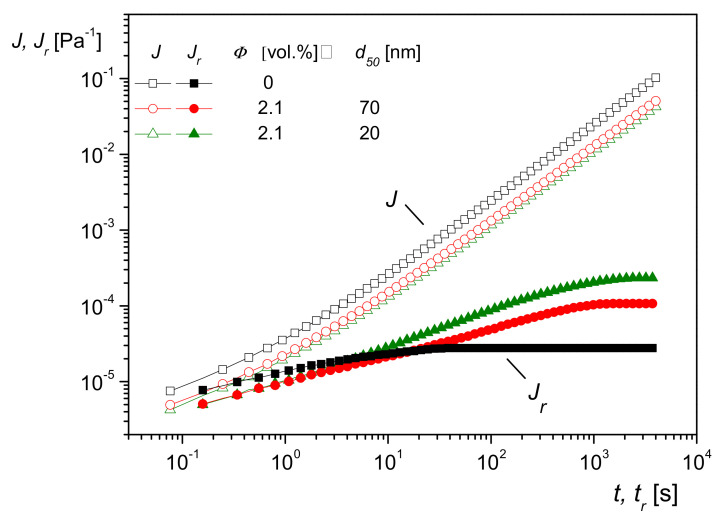
Creep compliance *J(t*) and creep recoverable compliance *J_r_*(*t_r_*) for the PMMA matrix and PMMA filled with 2.1 vol% of nanosilica of the mean diameters *d*_50_ = 20 nm and *d*_50_ = 70 nm, respectively, at 200 °C and shear stress of 50 Pa [22].

One consequence of the model sketched above is that the effect of molecules interacting with particles should become more pronounced the larger the specific surface area. This is shown to be valid in Figure 19, in which creep recovery experiments on PMMA filled with silica nanoparticles of the same composition and volume content but of two different sizes are plotted. Both curves for $J_{r}\left(t\right)$ lie above that of the matrix, as expected, but the sample with the smaller particle size and, consequently, the larger specific surface area exhibits the higher linear steady-state recoverable compliance. The influence of the different particle sizes on *J(t*), which is mainly determined by the viscous properties, particularly at longer creep times, cannot be seen within the accuracy of the measurements.

**Figure 20:**
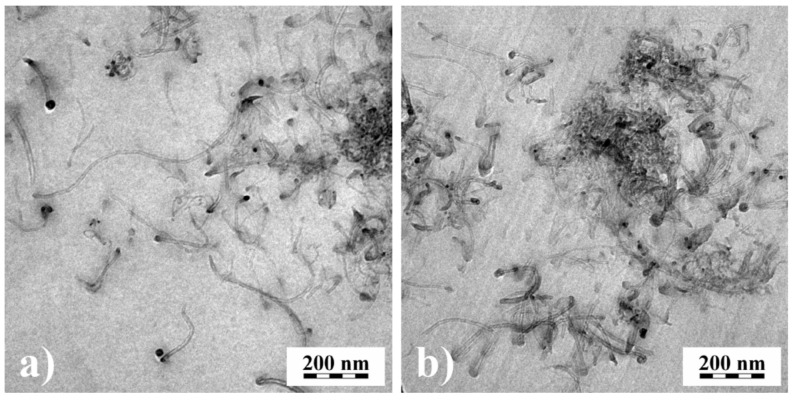
Transmission electron micrographs of PMMA filled with 0.6 vol% multi-wall carbon nanotubes (MWCNT) prepared in a solution of 15 wt% PMMA in dichloroethane using (**a**) magnetic stirrer (MS) or (**b**) high-speed stirrer (UT) [23].

The results on recoverable compliance lead to an interesting application of rheological measurements for getting information on the state of particle dispersion within filled systems. In particular, when easily agglomerating fillers are used, their good dispersion in a matrix is important for optimal efficiency. An example is rendering polymeric materials conductive by carbonic additives. The classical way of checking the distribution of nanosized fillers is transmission electron microscopy, which needs the preparation of ultrathin cuts. Figure 20 shows transmission electron micrographs of 0.6 vol% multi-wall carbon nanotubes (MWCNT) in PMMA prepared in a solution of 15 wt% PMMA in dichloroethane using a magnetic stirrer (MS) or a high-speed stirrer (UT). From the micrographs, it is hard to say which method has achieved the better distribution.

**Figure 21:**
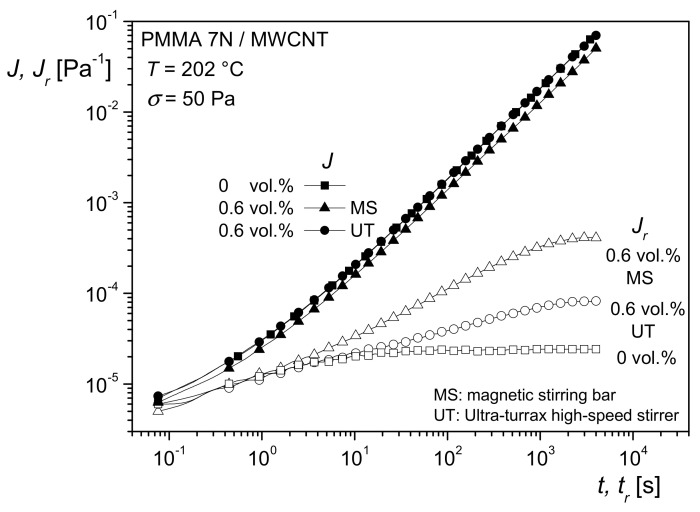
Creep compliance *J* as a function of creep time *t* and recoverable compliance *J_r_* as a function of recovery time *t_r_* for the PMMA matrix and PMMA with 0.6 vol% MWCNT prepared by using a magnetic stirrer (MS) or a high-speed stirrer (UT) [24].

Figure 21 shows the creep and recovery curves of the differently prepared samples. *J*(*t*) is approximately the same for the matrix and the two compounds. *J_r_*(*t_r_*) of the filled samples lies above that of the unfilled PMMA, but the plateau of the recoverable compliance is distinctly higher for the MS than for the UT sample. These differences of the filled samples can be interpreted by a larger specific surface area generated with the MS in comparison to the UT process, and, consequently, a better distribution of the MWCNT was obtained by magnetic stirring.

### 5.3. Viscosity Functions of Particle Filled Polymers

**Figure 22:**
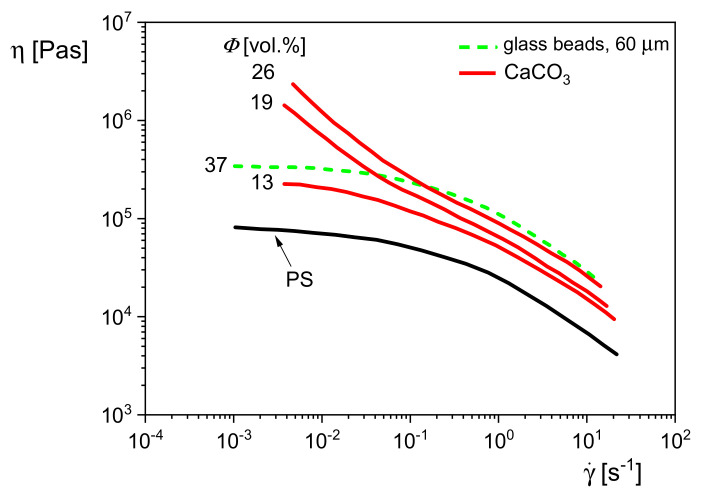
Viscosity functions of polystyrene filled with glass beads and calcium carbonate, respectively, at 190 °C.

Qualitative conclusions on structures built up by particles in polymer melts can be obtained from viscosity functions that describe the shear viscosity:

(15) $$\eta \;=\;\sigma /\dot{\gamma }$$

in dependence on the shear rate $\dot{\gamma }.$ *σ* is the shear stress. Viscosity functions are commonly used for an assessment of processing operations. Figure 22 shows the viscosity functions of polystyrene (PS) filled with glass beads or calcium carbonate (CaCO_3_), which have totally different geometries. The glass beads of a mean diameter of 60 μm and a volume concentration of 37% leave the shape of the curve of the matrix unchanged but shift it to higher viscosities. For the PS filled with the plate-like calcium carbonate of sizes smaller than those of the glass beads, a distinct change of the shape of the viscosity curve is already observed at 19 vol%. The viscosity increase with decreasing shear rate is due to interactions of particles, which can form structures that immobilize the matrix molecules to some extent, and even yield stresses may have to be overcome for flow. In the case of a yield stress *σ_y_*, the total stress *σ* can be written as:

*σ* = *σ_y_* + *σ_fl_* (16)

where *σ_fl_* is the stress of flow. For *σ_y_* >> *σ_fl_* Equation (16) becomes:

(17) $$\eta \;\approx \;\sigma_{y}/\dot{\gamma }\;\;\;\;\mathrm{or}\;\;\;\;\log\;\eta \;\approx \;\log\;\sigma_{y}\;-\;\log\;\dot{\gamma }$$

and from this relation *σ_y_* can be determined. In [21], the viscosity functions of several filled polymer melts are discussed, and it becomes obvious that interesting information about the structure built up by various fillers can be obtained from relatively simple rheological measurements.

Comparing the effects of the glass beads and the calcium carbonate in Figure 22, one may conclude that 19 vol% of the CaCO_3_ particles are already able to create an internal structure that leads to a yielding behavior, while the higher filler content of 37 vol% of the distinctly larger glass beads only increases the viscosity function of the polystyrene matrix due to hydrodynamics, but its shape remains unchanged.

### 5.4. Applications of Viscosity Functions for the Analysis of Structural Developments in Melts of Engineering Polymers

#### 5.4.1. Polyvinylchloride

**Figure 23:**
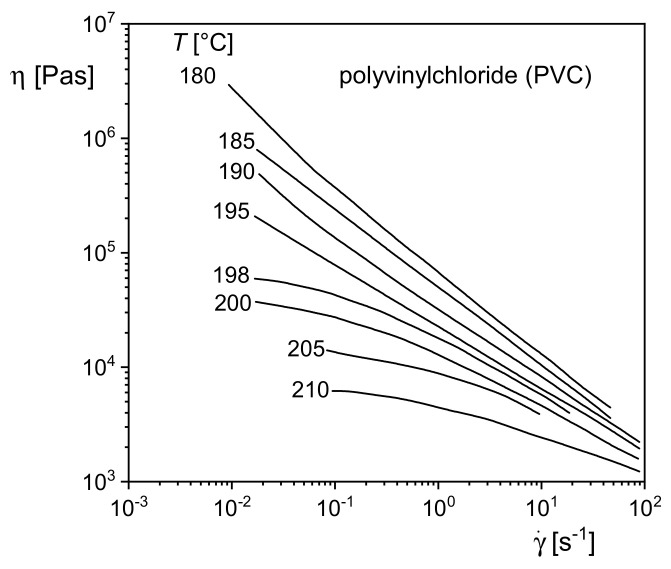
Viscosity *η* as a function of shear rate $\dot{\gamma }$ for polyvinylchloride (PVC) at various temperatures [26].

Figure 23 shows the viscosity functions of a commercial polyvinylchloride (PVC) at several temperatures between 180 and 210 °C. In contrast to a homogeneous polystyrene melt, for example, the shape of the viscosity functions changes with temperature. For temperatures up to 195 °C, the viscosities steadily increase with decreasing shear rates similar to the polystyrene filled with the larger contents of CaCO_3_ in Figure 22. For higher temperatures, however, the viscosity functions approach their zero-shear viscosities common for homogeneous polymer melts. Such behavior points to an internal structure existing within the PVC melt at lower temperatures but disappearing at higher ones. As can be seen from Figure 23, the change of shape of the viscosity functions occurs within a small range of temperature. This experimental finding supports the assumption that the structure is built up by crystallites still existing in the melt of PVC, which has been long known to be crystallizable (e.g., [25]). The influence of these structures on rheological properties was studied in [26], and their temperature dependence was correlated with the melting behavior of the crystallites. These results are of interest in the context of this paper because they show how usual measurements of viscosity functions can support the analysis of microscopic structures within a melt. Furthermore, studies of this kind on PVC support the optimization of its processability because crystallinity hinders the fusion of the PVC powder particles occurring in the polymerization process. Fused material is a precondition for the extrusion of items with good mechanical properties.

#### 5.4.2. Acrylonitrile-Butadiene-Styrene Copolymer

Acrylonitrile–butadiene–styrene copolymers (ABS) are widely used engineering plastics with excellent impact strength. They consist of a styrene–acrylonitrile (SAN) matrix and SAN-grafted polybutadiene (PB) rubber particles coupled to the matrix. The mechanical properties of parts from ABS are essentially affected by the amount of PB particles, the degree of grafting and their size and state of agglomeration. Particularly, the latter has raised frequent considerations because it deteriorates end-use properties and may be influenced by polymerization and processing conditions.

**Figure 24:**
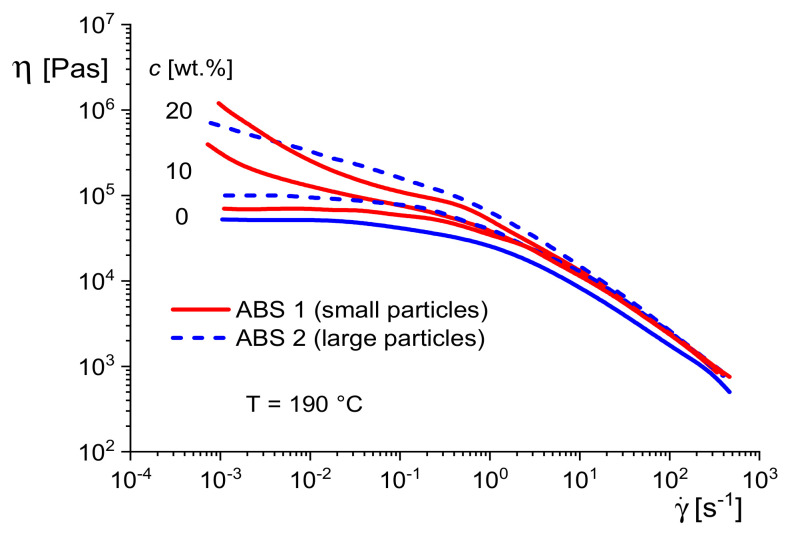
Viscosity functions of the styrene-acrylonitrile (SAN) matrix and acrylonitrile-butadiene-styrene copolymers (ABS) samples with small and large rubber particles at two concentrations each [27].

Figure 24 shows the viscosity functions of two ABS with the same rubber contents and those of their SAN matrices. For ABS 1, rubber particles with sizes of about 0.1 and 0.5 μm are dominant, and ABS 2 contains particles up to several microns. More detailed information on the morphologies can be found in [21].

For all samples, the viscosity increases by adding the rubber particles. However, the viscosity functions of the samples with the smaller rubber particles exhibit a viscosity increase at low shear rates for the two concentrations of 10 and 20 wt %, while for the sample with the larger particles, the shape of the viscosity function at 10 wt % is similar to that of the SAN matrix and a rise at lower shear rates occurs at 20 wt%, only. This result demonstrates once more the role of the specific surface area for the interaction between particles and matrix. At the same concentration, the smaller particles have a larger surface area, and the interaction with the matrix polymers can be more intensive, leading to a stronger reduction of their mobility.

**Figure 25:**
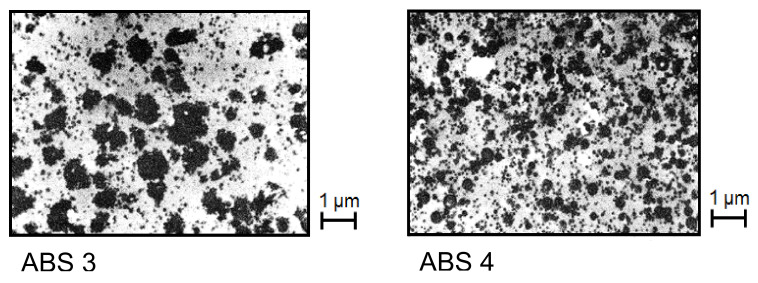
Electron micrographs of two ABS samples with different particle agglomeration. The SAN matrix and the rubber content are the same [27].

These relations can be used to get insight into the particle agglomeration of ABS. Figure 25 shows electron micrographs of two ABS samples with the same SAN matrix and rubber content but different states of particle agglomeration.

**Figure 26:**
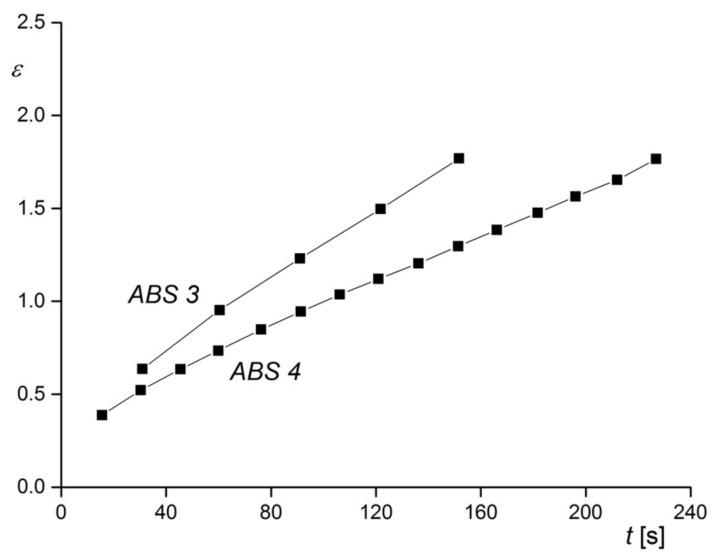
Hencky strain *ε* as a function of time *t* at a constant tensile stress of $\sigma_{E}\;$= 4.8 · 10^4^ Pa and 160 °C for ABS 3 and ABS 4 with different particle agglomerations shown in Figure 25 [27].

The rheological characterization was performed in elongation. This method is not so widely used as capillary or rotational rheometry and needs special equipment. Its advantage is that experiments are carried out free of walls in contact with the sample and, thus, slippage cannot occur, which may have to be considered for rubber-modified polymer melts. In Figure 26, creep experiments on the two samples of Figure 25 are shown. At the constant tensile stress chosen, ABS 3 with the larger particles shows the higher elongation as a function of creep time than ABS 4, and this behavior corresponds to the lower viscosity of ABS 3 (see Section 2.1.2). This example demonstrates how rheological measurements could be applied to trace the agglomeration in a polymer with rubbery fillers that may be exposed to different conditions during processing. For instance, the distribution of rubber particles can affect surface properties like the gloss of injection molded items. The advantage of rheological experiments is the relatively easy sample preparation in comparison to electron microscopy and the information obtained for a representative amount of a material instead for an only small section.

## 6. Conclusions

Rheological measurements are a powerful tool to detect structural changes within polymers. In some cases, they are distinctly more sensitive than analytical methods, but it is often impossible to draw any quantitative conclusions from the results. For this purpose, additional very specific techniques must be applied. For example, as well-known for common polymeric materials and demonstrated for linear polyethylenes in this paper, a power-law exists between the zero-shear viscosity $\eta_{0}$ and the weight-average molar mass $M_{w}$ and thus, small molar mass alterations are sensitively indicated by $\eta_{0}$. However, absolute values of $M_{w}$ and the molar mass distribution well must be determined by other methods like gel permeation chromatography coupled with laser light scattering; for example, If such a relation has been established for special material, it can be quantitatively applied to determine molar masses from measurements of the zero-shear viscosity. Long-chain branched species do not fulfill the power-law, but qualitative conclusions concerning their structure can be obtained from $\eta_{0}$ in dependence on $M_{w}$ if compared with $\eta_{0}$($M_{w}$) for linear samples.

The role of elastic rheological properties with respect to molecular analysis is limited insofar as the effects of molar mass distribution and long-chain branching is often difficult to separate. For a supporting role in the sensitive detection of long-chain branches, elongational experiments and, in particular, strain hardening can be used when an at least qualitative knowledge of the absence of high molar mass components exists.

The strong dependence of viscosity on molar mass can be used for studying the stability of polymeric material at various thermal or mechanical conditions. For practical purposes, even the melt index, which is easy to measure, provides valuable information.

Studies of thermorheological behavior provide interesting insights into the molecular uniformity of a polymeric material. Thermorheological complexity indicates a nonuniform composition of the material. In linear polymers, a blend of non-miscible molecules may exist; for branched species, molecules of different branching structures are probable. Thermorheological simplicity allows the calculation of activation energies that are polymer specific and, thus, can be used for analytical purposes.

For filled polymeric systems, rheological measurements react sensitively to interactions between particles and particles with matrix molecules. A particle network results in the occurrence of yield stress and frequency independence of the storage modulus. Filler/matrix interactions can be traced by an increase of the linear steady-state recoverable compliance. These effects may be used to study the state of agglomeration in dispersed polymeric systems at various treatments or even to follow-up the melting behavior of crystallites in a polymer melt.

## Data Availability

Not applicable.

## References

[1] Münstedt H., Schwarzl F.R. (2014). Deformation and Flow of Polymeric Materials.

[2] Liu C., He J., van Ruymbeke E., Keunings R., Bailly C. (2006). Evaluation of different methods for the determination of the plateau modulus and the entanglement molecular weight. Polymer.

[3] Münstedt H. (2014). Rheological experiments at constant stress as efficient method to characterize polymeric materials. J. Rheol..

[4] Stadler F.J., Piel C., Kaschta J., Rulhoff S., Kaminsky W., Münstedt H. (2006). Dependence of the zero shear-rate viscosity and the viscosity function of linear high-density polyethylenes on the mass-average molar mass and polydispersity. Rheol. Acta.

[5] Gabriel C. (2001). Influence of the Molecular Structure on the Viscoelastic Behavior of Polyethylene Melts. Doctoral Thesis.

[6] Onogi S., Masuda T., Kitigawa K. (1970). Rheological properties of anionic polystyrenes. I. Dynamic viscoelasticity of narrow distribution polystyrenes. Macromolecules.

[7] Resch J.A. (2010). Elastic and Viscous Properties of Polyolefin Melts with Different Molecular Structures Investigated in Shear and Elongation. Ph.D. Thesis.

[8] Münstedt H. (2019). Elastic Behavior of Polymer Melts.

[9] Gabriel C., Münstedt H. (2002). Influences of long-chain branches in polyethylenes on linear viscoelastic flow properties in shear. Rheol. Acta.

[10] Wood-Adams P., Costeux S. (2002). Thermorheological behavior of polyethylene: Effects of microstructure and long chain branching. Macromolecules.

[11] Van Gurp M., Palmen J. (1998). Time temperature superposition for polymeric blends. Rheol. Bull..

[12] Kessner U., Münstedt H. (2010). Thermorheology as a method to analyse long-chain branched polyethylenes. Polymer.

[13] Stadler F.J., Chun Y.S., Han J.H., Lee E., Park S.H., Yang C.B., Choi C. (2016). Deriving comprehensive structural information on long-chain branched polyethylenes from analysis of thermo-rheological complexity. Polymer.

[14] Chen C., Shekh M.I., Cui S., Stadler F.J. (2021). Rheological behavior of blends of metallocene catalyzed long-chain branched polyethylenes. Part I: Shear rheological and thermorheological behavior. Polymers.

[15] Auhl D., Stange J., Münstedt H., Krause B., Voigt D., Lederer A., Lappan U., Lunkwitz K. (2004). Long-chain branched polypropylenes by electron beam irradiation and their rheological properties. Macromolecules.

[16] Münstedt H. (2011). Rheological properties and molecular structure of polymer melts. Soft Matter.

[17] Hanson D.E. (1969). Shear modification of polythene. Polym. Eng. Sci..

[18] Rokudai M., Mihara S., Fujuki T. (1979). Influence of shearing history on the rheological properties and processability of branched polymers. II. Optical properties of low-density polyethylene blown films. J. Appl. Polym. Sci..

[19] Rokudai M. (1979). Influence of shearing history on the rheological properties and processability of branched polymers. J. Appl. Polym. Sci..

[20] Triebel C. (2012). Rheologische Eigenschaften nanopartikelgefüllter Polymerschmelzen (Rheological Properties of Nanoparticle Filled Polymer Melts). Ph.D. Thesis.

[21] Münstedt H. (2016). Rheological and Morphological Properties of Dispersed Polymeric Materials.

[22] Münstedt H., Köppl T., Triebel C. (2010). Viscous and elastic properties of poly(methyl methacrylate) melts filled with silica nanoparticles. Polymer.

[23] Triebel C., Katsikis N., Stara H., Münstedt H. (2010). Investigations on the quality of dispersion of nanofillers in poly(methyl acrylate) composites by creep recovery experiments. J. Rheol..

[24] Katsikis N. (2008). Particle-Polymer Interactions in Melts of Nano- and Microcomposites with poly(methyl methacrylate) as Matrix. Ph.D. Thesis.

[25] Rehage G., Halboth H. (1968). Röntgenographische Untersuchung der Kristallisationsfähigkeit von Polyvinylchlorid. Makrom. Chem..

[26] Münstedt H. (1977). Relationship between rheological properties and structure of polyvinylchloride. J. Macrom. Sci. Phys..

[27] Münstedt H. (1981). Rheology of rubber-modified polymer melts. Polym. Eng. Sci..

